# Reframing the challenges to integrated care: a complex-adaptive systems perspective

**DOI:** 10.5334/ijic.843

**Published:** 2012-09-18

**Authors:** Peter Tsasis, Jenna M Evans, Susan Owen

**Affiliations:** School of Health Policy and Management, Faculty of Health, York University, 4700 Keele Street, Toronto, Ontario, Canada M3J1P3; Institute of Health Policy, Management and Evaluation, Faculty of Medicine, University of Toronto, 155 College Street, Suite 425, Toronto, Ontario, Canada M5T3M6; National Healthcare Advisory, KPMG Canada, 333 Bay Street, Suite 4600, Toronto, Ontario, Canada M5H2S5

**Keywords:** health systems integration, integrated care, complex adaptive system, partnership, inter-organizational, inter-professional

## Abstract

**Introduction:**

Despite over two decades of international experience and research on health systems integration, integrated care has not developed widely. We hypothesized that part of the problem may lie in how we conceptualize the integration process and the complex systems within which integrated care is enacted. This study aims to contribute to discourse regarding the relevance and utility of a complex-adaptive systems (CAS) perspective on integrated care.

**Methods:**

In the Canadian province of Ontario, government mandated the development of fourteen Local Health Integration Networks in 2006. Against the backdrop of these efforts to integrate care, we collected focus group data from a diverse sample of healthcare professionals in the Greater Toronto Area using convenience and snowball sampling. A semi-structured interview guide was used to elicit participant views and experiences of health systems integration. We use a CAS framework to describe and analyze the data, and to assess the theoretical fit of a CAS perspective with the dominant themes in participant responses.

**Results:**

Our findings indicate that integration is challenged by system complexity, weak ties and poor alignment among professionals and organizations, a lack of funding incentives to support collaborative work, and a bureaucratic environment based on a command and control approach to management. Using a CAS framework, we identified several characteristics of CAS in our data, including diverse, interdependent and semi-autonomous actors; embedded co-evolutionary systems; emergent behaviours and non-linearity; and self-organizing capacity.

**Discussion and conclusion:**

One possible explanation for the lack of systems change towards integration is that we have failed to treat the healthcare system as complex-adaptive. The data suggest that future integration initiatives must be anchored in a CAS perspective, and focus on building the system’s capacity to self-organize. We conclude that integrating care requires policies and management practices that promote system awareness, relationship-building and information-sharing, and that recognize change as an evolving learning process rather than a series of programmatic steps.

## Introduction

For over two decades, health services researchers and managers have focused on the integration of care as a means to improve system performance and the patient experience. The delivery of integrated care involves coordinating services across multiple healthcare professionals, organizations, and sectors, and prioritizing patient needs and preferences [1]. Aging populations, the growing burden of chronic disease, new technologies and treatments, and financial constraints have led healthcare systems around the world to seek fundamental changes in system design with integration strategies as a principal feature of reform efforts [2].

For example, Ontario, the most populous province in Canada, introduced a novel governance model in 2006 with the development of the Local Health Integration Networks (LHINs). The fourteen geographically defined LHINs were mandated by the Ontario Ministry of Health and Long-Term Care to better integrate healthcare services using integrated health service plans developed collaboratively with local healthcare providers and community members [3]. With the advent of the LHINs, health service providers in Ontario are challenged to select and manage partnerships that optimize the delivery of high quality, cost effective, patient-centred care [4].

More than five years have passed since the LHINs took on their full authority, and “serious problems with how patients move through the healthcare system, from the emergency department to hospital to long-term care” persist [5]. Even though Ontario has experienced some notable successes in improving integration and the patient care experience with initiatives such as the Aging at Home Strategy and the Regional Cancer Program [6, 7], the reactions of providers and patients, and the results of ongoing monitoring and measurement, reveal that the healthcare system continues to function in silos, falling short of expectations [8]. Canada, and the province of Ontario, rank worse than many other major countries and jurisdictions in the timely transfer of information across health services providers and in wait times; both problems have been attributed to a lack of integration [5, 9].

The Ontario experience, however, is not unique. Developing successful and sustainable integrated systems remains an ongoing global challenge [10–12]. Multiple definitions and models of integrated care exist [13] ranging from the traditional, single ownership “integrated delivery system” [14] to more flexible arrangements based on partnership contracts [15]. Specific features and experiences of integrating care also vary by social, political and economic context. Nevertheless, the multi-level enablers and barriers to the collaborative work necessary for integration are strikingly similar across healthcare systems. Recent reviews identify the following key success factors: shared financial and clinical information systems; physician participation; interprofessional teamwork; compensation models that support collaboration; transformational leadership to bridge divergent cultures and promote the vision of integrated care; performance measurement, reporting and rewards associated with system-level cost and quality outcomes; and governance and policy structures for shared accountability [16–20]. These integration prerequisites are well known and documented, even in early seminal work on integrated delivery systems [14, 21], which begs the question, why has systems integration not developed widely in light of growing empirical work, existing methods and ongoing efforts to integrate care?

We hypothesized that part of the problem may lie in how we conceptualize the integration process and the complex systems within which integrated care is enacted. To date, integration researchers have applied theories of organizational culture, change, strategy, performance, leadership, and design [22, 23], networks [24, 25], structure and agency [26], institutionalism [27, 28], and organizational ecology [29]. The resulting body of knowledge has informed our understanding of the structures, processes, outcomes, and experiences of integrated care. Increasingly, however, scholars are advocating for the application of complex-adaptive systems theory to integrated care [30–32].

Complex-adaptive systems (CAS) are open systems with fuzzy boundaries comprised of numerous, diverse and highly interactive agents [33]. Their patterns of interaction and ongoing adaptations often contribute to novel and unpredictable behaviours and events; CAS are thus characterized as emergent and self-organizing [33]. Although CAS theory has been used to describe healthcare organizations and inform health services and policy research for at least ten years [34–42], the potential value of a CAS perspective to health systems integration has only recently been realized and explored, and empirical work in the field is limited [30–32].

Nugus et al. argue that a linear ‘continuity of care’ view of the patient journey must be supplemented by a complex-adaptive lens to help explain non-linearity and the emergent behaviours of multi-level dynamic networks of actors [32]. Drawing from an ethnographic case study of integration in the emergency department, they characterize integrated care as “managing the patient trajectory in the porous, shifting and negotiable boundaries of health services” (p. 2002) [32]. In a conceptual paper, Edgren and Barnard apply CAS principles to integrated care to explain the adverse effects of top-down leadership practices which reduce actors’ motivation and ability to innovate [31]. Instead, they argue, managers must work as facilitators to create the conditions that enable self-organizing [31]. Adopting a CAS lens, therefore, contributes to different ways of thinking about not only the healthcare system as a whole, but also the patient journey, the role of leaders, and the very process of integrating care.

This study aims to contribute to discourse regarding the relevance and utility of a CAS perspective on integrated care. For the purposes of this study we define integration broadly as the process of multiple professionals, organizations and sectors collaborating and implementing changes to provide coordinated patient care. We draw from a series of focus groups held in the Greater Toronto Area of Ontario, Canada with a range of healthcare providers and managers involved in integration initiatives. The purpose of the study was two-fold: (1) to understand how healthcare professionals perceive the context for integration, including their views and experiences of the healthcare system and of health system change towards integration, and (2) to identify and understand, based on their experiences, the key factors influencing partnerships aimed at integrating care. We use a CAS framework to describe and analyze the data, and to assess the theoretical fit of a CAS perspective with the dominant themes in participant responses.

## Methods

A focus group approach to data collection was undertaken with ethics approval from the York University Office of Research Ethics. Focus groups provide an effective means for exploring and understanding the social context within which healthcare professionals’ experiences are embedded, while giving the opportunity for individual perspectives to be challenged or further explored through interaction [43]. The period of recruitment was from February to June 2009. The sample consisted of 36 participants and began as a convenience sample of self-identified healthcare professionals from the Central LHIN with experience in health systems integration and partnership formation. This sample was expanded through snowball sampling to include participants from across the Greater Toronto Area. All participants held boundary spanning roles, representing their organization within the healthcare system, and thus could provide us with their unique and information-rich experiences and insights on the topic of inquiry.

The participants represented a broad range of health services organizations spanning the healthcare continuum from primary care (n=4), acute care (n=10), long-term care (n=4), home and community support (n=13) and the LHINs (n=5). Their professional training was diverse and included nursing (n=6), medicine (n=5), social work (n=3) and management (n=22). A total of six focus groups were held, consisting of 6–8 participants per group. Participants were selected for each group to ensure group heterogeneity in the organizations and professions represented. Various experiences and perceptions facilitate discussion and enable investigators to capture points of consensus and disagreement [43].

Each focus group discussion lasted for approximately two hours. Two investigators served as moderators and a third took field notes. A semi-structured interview guide was used to facilitate discussion using open-ended questions. The interview guide consisted of two sections, one on the system context in relation to integration and the other on partnership work in relation to integration. Questions on the system context sought participant views on system characteristics, system priorities, and enablers and barriers to system change and improvement. Questions on partnership work sought participant feedback on the enablers and barriers to partnership formation and success. Emphasis was placed on personal experiences and story-telling. In addition, what was said was explored further in subsequent focus group sessions by probing to uncover perceptions or ideas that had not been anticipated at the outset of the research. Additional questions were thus added to the interview guide as the researchers became familiar with the issues discussed within the focus of the inquiry. For example, participants were asked to comment on levels of engagement and on the sustainability of the initiatives they have been involved with to date. At the end of each focus group, the key themes or points recorded in the field notes were reiterated back to participants to allow them to make corrections or additions they deemed necessary, as a form of member checking. At this time, participants also had the opportunity to make any final comments on issues or concerns that they felt were not addressed during the session. The data collection process was terminated when similar discussions began to emerge and no additional names of potential focus group participants for the study came forth.

All focus group discussions were audio-taped and transcribed verbatim. The transcripts were verified to identify omissions or errors. Data were coded and categorized through the use of content analysis independently by three investigators [43]. Commonalities and differences were noted, along with saliencies in the data such as patterns across experiences and perspectives, in the development of overarching themes. These themes were validated by triangulation through convergence among responses by the three investigators and organized into four broad categories representing the key challenges to integration: (1) system complexity, (2) inter-organizational and inter-professional ties and alignment, (3) funding mechanisms, and (4) a command and control environment. Each of these categories consisted of multiple sub-themes. For example, under “inter-organizational ties and alignment” are the following sub-themes: (a) culture and language, (b) relationship-building and trust, (c) time and resources, (d) shared vision, and (e) shared leadership and accountability.

Quotes from the focus groups were compiled under each theme in order to identify quotes most illustrative of the views of the participants. The results were then reviewed for their relevance to the key characteristics of CAS and re-organized under three broad headings to allow for discussion and analysis of their meaning using a framework of characteristics of CAS: (1) diverse, interdependent and semi-autonomous actors, (2) embedded co-evolutionary systems, emergent behaviours and non-linearity, and (3) self-organizing capacity and simple rules. The framework we used of CAS characteristics consists of the most commonly identified descriptors in the scholarly literature as identified by a review and concept analysis of CAS definitions [44].

## Results

### Diverse, interdependent and semi-autonomous actors

CAS consist of a large number of diverse and interdependent components. Participants recognized this complexity in the healthcare system: “It’s such a complex system and there are such different players” (Manager, Acute Care, FG5). In addition to the general divide between acute care and community-based care, participants described a range of different cultures and languagesf based on organization type (hospital, long-term care home, community health centre, mental health agency, home care association, etc.) and professional group (managers, physicians, nurses, pharmacists, social workers, policy-makers, etc.). This diversity can contribute to creativity and problem-solving, but is often also a source of communication and coordination difficulties [45]. For example:

“You may have three or four organizations supporting a family in a community...how do you have them speaking the same language and sharing information at the local level?” (Social Worker, Home and Community Support, FG6).

Participants identified as a barrier to integration the limited awareness and understanding across organizations and professionals of what various components of the system do and how they are linked:

“Even though we are working in the same community, we don’t necessarily understand exactly what other organizations do and how they contribute to the care of the client. Everyone gets focused on their own respective area and we become myopic” (Nursing Manager, Acute Care, FG4).

This notion of understanding the system and the respective roles of those working in the system to support collaborative activities mirrors Batalden and Mohr’s concept of “knowledge of a system” [46]. Batalden and Mohr suggest that healthcare leaders and staff must first understand the system’s services, customers, processes, inputs, and suppliers in order to develop a shared vision for the future and a viable plan for improvement [46]. Similarly, one participant noted that those involved in integrating care must have a broad understanding of the different parts of the system so they may “communicate what it’s for, when it’s applicable, and how to get at it” (Social Worker, Home and Community Support, FG4). This understanding, another participant explained, “makes integration easier and you can’t necessarily have that mandated because it goes back to relationship-building and trust” (Manager, LHIN, FG3).

The importance of relationships was a recurrent theme in the data. Participants emphasized the lack of opportunity and time to forge sustainable relationships with other organizations in order to better understand their interdependencies and collaborate to deliver integrated care. For example:

“You can have four, five, ten agencies at the table, and you haven’t put in that time to understand the agencies, their culture and the environment, so that you can build a working relationship” (Nursing Manager, Acute Care, FG2).

Without substantive relationships and heedful interactions, developing a shared vision and coordinating actions for integrated care is difficult [47] and can easily be neglected as the following two participants argued: “There needs to be buy-in from all the parties and that takes some time. That takes dialogue” (Manager, LHIN, FG6) and “I think we just jumped in ... we were not strategic and there was no vision set out” (Manager, Home and Community Support, FG1). Participants observed that the development of a shared vision often does not occur in practice, in part because a common question circulating in the field is, “what does integration really mean?” This lack of clarity suggests the need for more up-front dialogue towards identifying preferred methods and desired outcomes of integrated care and work to build consensus, or “shared mental models” around such issues within system boundaries [48]. This would help alleviate the fear and anxiety participants have observed in integration planning: “There is so much anxiety and when there’s anxiety, people dig their heels in because ‘I am not going to give anything up because I don’t know what I’m going to get’…and you don’t want to be consumed by another organization” (Manager, Home and Community Support, FG1). Participants accepted that diversity in goals and practices across organizations and professionals is an inherent reality of healthcare delivery: “we have to give latitude and accept that as long as they’re contributing to the shared vision” (Manager, LHIN, FG6).

In general, the participants’ experiences confirm a high level of diversity and interdependence among health system stakeholder groups, particularly in the context of integration initiatives. Boundaries between organizations and professional groups are strongly felt through differences in culture and language, but are also porous, allowing for exchange, movement and learning. While the various actors and elements of the system recognize their interdependencies, their interactions are often not of the quality and frequency that support the delivery of integrated care. Our data suggest two key reasons for this. First, limited awareness, knowledge and understanding of the system and its various components magnify differences and confusion among groups. As one participant so aptly put it:

“Confusion begets resistance and works against trust because if people are confused about their roles or confused about what’s going to happen next or confused about the goal, then they’re going to be insecure and less able to trust” (Physician, Primary Care, FG5).

Second, a lack of time and resources restrict opportunities for ongoing relationship-building. These limiting conditions are created and shaped by environmental factors (which we discuss further below), revealing that actors’ attitudes, capabilities, and behaviours regarding integration are not completely self-directed.

### Embedded co-evolutionary systems, emergent behaviours and non-linearity

CAS reside within and interact with other systems in a nested or embedded fashion. Due to complex interdependencies and ongoing interactions among elements within and across embedded CAS, new (or emergent) behaviours are common represented by constant co-evolutionary adaptations [39]. Because elements change and behaviour is emergent in CAS, cause and effect relationships are not directly evident or linear. The trajectory of the system is thus unpredictable, though general patterns are discernible [39].

Participants recognized the embeddedness of the healthcare system within larger social systems: “Healthcare is a small component of actually making a population healthy. It’s socioeconomic factors, it’s education, it’s social. There are twelve determinants of health” (Social Worker, Home and Community Support, FG6). Another participant argued that promoting health “requires more than the Ministry of Health and Long-Term Care, more than the Ministry of Health Promotion. It’s important we have those communications with sectors outside of health” (Manager, Home and Community Support, FG3). Each community, represented by the geographic boundaries of the LHINs, as well as networks consisting of two or more organizations, individual organizations, and even departments and programs, can also be considered CAS, demonstrating the complexity and degree of embeddedness of healthcare delivery. For example:

“There are a lot of levels you’re working down before you really see what’s happening on the front-line. It’s hard to envision how things actually roll out at the client level because something can sound great in a proposal and make complete sense but it’s a great challenge when you get down to those finite details” (Manager, Acute Care, FG6).

This quote also suggests the potential for unexpected events despite well-planned and well-intentioned interventions. Participants expressed frustration at the lack of systems change despite ongoing efforts, demonstrating the non-linearity inherent in integration work:

“I found in the beginning people were coming out in droves to the meetings...but then we weren’t necessarily seeing things come to fruition. People were getting frustrated and we started to see less and less of them…it seems like we are doing all these efforts and things aren’t necessarily going through” (Manager, LHIN, FG2).

“You know everybody is willing…they want to work together, they want to make it work and the system makes you disinclined to actually push that through” (Manager, Long-Term Care, FG2).

Participants also noted that interventions to integrate care may be sub-optimal or fail as a result of misalignment or trade-offs among the various elements in the system—an example of the principle of co-evolution. This notion is best expressed through the words of one participant who said:

“So what is it that you have to go back and change as a result of the change you’re making? What are the interdependencies? There are other things that this change impacts on that we need to address to support the change. If we don’t do that and we don’t realize we’ve got something over here that’s dragging things back, at the same time we’ve got something that’s trying to mobilize it forward, people get caught in the middle” (Manager, LHIN, FG5).

In addition to CAS being influenced by other external and internal systems, they are also history-dependent, their current behaviours being shaped (but not necessarily determined) by their past [49]. History was a key barrier identified by participants who said:

“You want us to dismantle what we’ve built up for years and now come together with multiple partners where there was a lot of competition before” (Manager, Long-Term Care, FG6)

“We’re talking about two different cultures, an acute culture and a community culture…there’s a lot of barriers just because of the history” (Manager, Home and Community Support, FG4).

Participants also discussed the importance of and challenges involved in balancing conflicting needs. The notion of health systems integration and management as a ‘balancing act’ resurfaced numerous times in the discussions; participants noted the need to balance: (a) standardization with innovation, (b) accountability with flexibility, (c) order and direction from above with participation and buy-in from below, (d) organizational needs with system needs, and (e) collaboration with healthy competition. In CAS, such tensions and paradoxes are natural, cannot necessarily be resolved, and may in fact work together in positive ways [39]. In general, participant views and experiences integrating care are in alignment with the principles of CAS as embedded, co-evolving, emergent, non-linear and unpredictable.

### Self-organizing capacity and simple rules

In CAS, order is emergent through a process of self-organization, and maintained without central control [39]. Efforts to exert control are usually futile and can be intrusive, slowing down the capacity of the system to react and adapt [49]. Rather than top-down control and rigid structures, the use of broad guiding principles, or simple rules, should be used to encourage change in the desired direction without stifling creativity and innovation [49]. CAS are sensitive to small changes [50], evoking the principle of non-linearity and demonstrating the power of simple rules.

Participants provided a variety of examples that demonstrate ways in which the self-organizing capacity of the healthcare system has been restrained by rules that are anything but simple. For example, through funding policies that support only ‘medically necessary’ hospital and physician services, the Canada Health Act restricts funding integration, making it difficult to link health services across boundaries in support of patient care pathways. The lack of community control in current funding approaches was made explicit by the following participant statement:

“…we don’t have the mandate to be able to steer the funds in what would be more impactful at the local level. It still comes from Queen’s Park (Ontario Ministry of Health and Long-Term Care), very much command and control” (Manager, LHIN, FG5).

To support the delivery of integrated services, differentiated funding based on service type or provider must be replaced by the pooling of funds across services and sectors [20, 51–53]. According to participants, limited funding and inadequate funding mechanisms represent a major barrier to integrated care partnerships: “Part of the challenge is that integration is being done essentially on the sides of people’s desks!” (Manager, Home and Community Support, FG4) and the LHINs “just give out a funding call for applications and then expect that overnight, agencies can come together and develop the kind of culture and set of values for working together” (Manager, Home and Community Support, FG5). Other participants elaborate further:

“…there’s a lot of funding that goes into the community, but it’s not integrated. It’s hugely limiting in many ways in terms of how you partner and who you can partner with” (Manager, Home and Community Support, FG3).

“It’s not a level playing field and it never has been. Hospitals are dealt with differently than the community. The problem lies within the community as well. The smaller organizations that may have culturally-specific services are not seeing the funding that these mega community service agencies are starting to build on” (Manager, Long-Term Care, FG1).

According to participants, key features of an effective funding strategy—one that incents rather than inhibits collaborative behaviours—include more equitable access to resources across organizations and sectors, flexibility in how funds may be used, and a stronger patient focus.

In addition to the adverse effects of existing funding approaches, participants argued that legislation and policy require modification to allow organizations and providers to engage fully in integration efforts and to reduce the potential influence of political agendas on the process. For example:

“There’s the legislation, how things are planned, how policy is made in government…when we’re trying to come up with solutions in our healthcare system sometimes there’s just a lot of bureaucracy. So it is hard to have an integrated system” (Manager, LHIN, FG2).

“That’s something I’ve found to be a disincentive because the legal requirements that are being imposed for fear of risk are just so overwhelming” (Manager, Long-Term Care, FG3).

Participants further explain that although the LHINs represent a viable effort to instigate integration, their impact has been limited by what many of them deem a “command and control environment.” For example:

“They [the LHINs] have already been told by the province what the three priorities are that have to go into the integrated health service plans, but those priorities are not strategic nor systemic. They are reactionary priorities.” (Nursing Manager, Acute Care, FG2).

“The legislation hasn’t kept up with the mandates of the LHINs around integration so there’s a lot of barriers...Their hands are really tied as to what they can truly offer” (Manager, Home and Community Support, FG5).

Another example of the impact of historical policy barriers and what one participant called “conflicting infrastructures” (Manager, Home and Community Support, FG3) is the lack of physician-system integration. Primary care is considered an integral building block of integrated health delivery, but physicians often lack leadership roles in the design and implementation of integrated care processes [20, 52]. Without the engagement and systematic involvement of physicians in integration initiatives, progress is limited:

“If the LHINs want to make a difference, they can’t because primary care is in the mandate of the province. That hinders the positive change LHINs want to make” (Manager, Acute Care, FG1).

While much of the focus group discussions supported the importance of fostering the self-organizing capacity of the healthcare system rather than stifling it with central control, participants still emphasized the role of external facilitation:

“…external facilitation may be the key to some of these things because if one partner takes over and drives the process, they look aggressive and nobody trusts them” (Manager, Home and Community Support, FG3).

This quote suggests that there is a distinction between “command and control” leadership and indirect or facilitative leadership [30]. Furthermore, like the CAS principle of distributed, rather than central, control, participants emphasized the importance of shared leadership and shared accountability:

“I think the big piece of it is leadership and whether it’s shared leadership of the initiative …You have to have a good leader, somebody who is able to not necessarily be the expert, but to be able to work with everyone” (Nursing Manager, Acute Care, FG1).

“It’s about shared accountability so no one feels as though they’re the only ones leading it or that they are the only ones that have to come up with the solution. There’s that real sense of sharing the work that needs to be done” (Manager, Long-Term Care, FG2).

In summary, participants strongly emphasized the influence of contextual factors on their efforts and their ability to integrate care, which highlights the interplay and tensions between structure and agency [26] and aligns with the CAS view of actors as semi-autonomous, events as history-dependent, and over-control as counter-productive.

## Discussion

This study’s focus group participants exposed a complex set of barriers to achieving the vision of integrated care in Ontario. Despite the efforts of the LHINs and varied local service providers, integration is challenged by system complexity, weak ties and poor alignment among professionals and organizations, a lack of funding incentives to support collaborative work, and a bureaucratic environment based on a command and control approach to management. Participant descriptions of the healthcare system and their integration experiences reflected many of the characteristics of CAS, including the presence of a wide variety of interacting elements, an inherent unpredictability that makes planning and alignment difficult, and the need to understand interdependencies, including points of apparent contradiction. In general, this study supports previous work that argues for a CAS perspective on integrated care [30–32].

Many of the barriers and enablers to health systems integration identified in this study are well-documented in the literature [16–20]. However, using a CAS lens to interpret our findings helped us reframe these barriers to reveal new insights. Our data suggest that one possible explanation for the lack of systems change towards integration is that we have failed to treat the healthcare system as complex-adaptive. In other words, barriers to the delivery of integrated care may be associated with reductionism and determinism under the scientific management paradigm in which inputs are transformed into outputs. Framing the healthcare system in this ‘machine’ metaphor perpetuates the belief that change is linear and predictable, and that it can be achieved through orderly planning and control processes.

CAS, as an alternative metaphor, frames healthcare delivery as a complex system that changes when the conditions are favourable through self-organization. In CAS, change cannot be forced and attempts to control the system or prescribe innovation are often counterproductive due to the potential to destabilize the system [49]. Instead CAS require “direction without directives” [35]. In other words, to achieve integrated care, an environment must be created that fosters connectivity among health services providers and organizations. Through ongoing interactions over time, creative solutions, such as new ways of organizing, will emerge based on collective insight, distributed control, and learning [31, 49]. This ‘hands-off’ approach is in stark contrast to most current interventions to improve integration which use direct authority to impose change rather than fostering and supporting collaboration from the bottom-up. In fact, Kodner and Spreeuwenberg argue that past integration efforts have failed to effect change at the service delivery level because they have been top-down approaches [54]. Barnsley et al. further suggest that facilitative leadership fosters learning in integrated delivery systems [55]. Under a facilitative leadership model, leaders remove obstacles and establish open communication channels, while organizations and professionals determine how to best achieve system goals.

Traditional strategic planning has oversimplified or misrepresented the complexity of the system. Relationships among the various health and social service organizations in the system are not well-understood thereby contributing to tugs-of-war when interventions are initiated and implemented to pull the system in one direction, while competitive forces elsewhere in the system are pulling in the opposite direction. Furthermore, healthcare policies fail to instigate relationship-building, trust, and alignment across organizations and professional groups. Begun et al. argue that unsuccessful integration initiatives result from “overstructuration and over-control in an uncertain and dynamic environment” [49]. Our data support this observation and suggest limited self-organizing capacity exists in the Ontario healthcare system as a result of overstructuring and over-control.

Health system reform towards integration in Ontario may require incremental modifications to policy and legislation in order to create a context that supports, rather than restrains, self-organization. Such efforts can begin with attention to fundamental issues raised by this study’s focus group participants, including sharing of information and knowledge, and fundamental changes to how care is funded. Funding must follow the patient, support collaboration across services and sectors through pooled budgets, ensure equitability, and encourage physician involvement. According to CAS theory, changes such as these will create an environment that facilitates cooperation among health service professionals and influence behavioural change in the system more effectively than directives that command organizations and providers to collaborate [31]. Some top-down actions will be required as well, to encourage widespread implementation, adequate evaluation, and the sustainability of integration initiatives [56, 57]; however, this work must be focused on modifying contextual factors that influence the system’s capacity to integrate with a focus on flexible structures that foster a level of interdependence that is neither too loose nor too tightly coupled [30].

At the meso-level, identifying and understanding the roles and activities of others in the system is a practical starting point for organizations seeking to partner with other providers to deliver integrated care. System awareness forces an organization to re-examine its own role and reconsider its vision and approach to organizing and managing its activities, information and resources. Interdependencies within the system require that time and resources be dedicated to bringing inter-professional groups to the table to support the assimilation of distinct professional cultures, and their languages and ideologies, to developing long-term working relationships rooted in a shared vision, and to building communication pathways that allow best practices and technological advances to flow across the care continuum [31]. As more and more organizations and professionals engage in this knowledge-building process and make relevant adjustments to practice that consider organizational interdependencies and relative contributions, new ideas and opportunities can emerge. CAS theory suggests that—given the right conditions—the healthcare system can and will self-organize into meaningful partnerships using its inherent competencies [30]. The principle of self-organization also suggests that once partnerships are formed, they need not be static. New partnerships can emerge simultaneously as old ones are dismantled, depending on the demands of the environment and the challenges it imposes.

## Limitations

This study used a relatively small convenience and snowball sample of healthcare professionals from the Greater Toronto Area. The transferability of the findings to other settings is limited by contextual differences in policy, governance, funding, history and patient populations as well as potential differences in the characteristics of the participants compared with others who were not identified for inclusion. Future research should examine how healthcare professionals in other contexts perceive systems integration and partnerships and the extent to which their experiences align with the principals of CAS theory. Furthermore, this study was exploratory in its focus; further work is needed to validate the applicability and relevance of a CAS perspective on integrated care and the implications for associated management practices. Finally, not all problems and desired changes in healthcare lie “in the zone of complexity”, suggesting that a CAS perspective is not always relevant or best applied in isolation [39]. Our data suggest, however, that a CAS perspective is both appropriate and useful as a framework for conceptualizing and guiding the delivery of integrated care.

## Conclusion

This study began with the aim to better understand why integrated care has not developed widely among Ontario service providers despite existing strategies and ongoing efforts. Based on their personal experiences and observations, focus group participants revealed complex interdependencies, relationships and contradictions in the healthcare system that suggest that healthcare delivery is a CAS that defies traditional paradigms and approaches to management. Considering the ongoing challenges in achieving integrated care in many international healthcare systems, we must question existing theoretical frameworks and management approaches. CAS theory provides an alternative and potentially useful mental model for conceptualizing health systems integration.

A CAS perspective advances our understanding of integration in the Ontario context by drawing our attention to interactions and relationships in the system rather than parts in isolation. By shifting our focus in this way, we identified the system’s limited capacity to self-organize as a key underlying barrier to integration. Future initiatives aimed at promoting or studying systems integration can benefit from the application of a CAS lens in combination with other theoretical frameworks to form more comprehensive and accurate models of how the healthcare system functions and how change initiatives, such as integrated care, may be achieved. Health systems integration requires policies and management practices that support relationship-building and information-sharing across organizational and professional boundaries, and that recognize change as an evolving learning process rather than a series of programmatic steps. Future research may explore how knowledge and beliefs relevant to the delivery of integrated care are exchanged and transferred (or not) vertically and horizontally under different contextual conditions. This work should (a) build on recent advances in our understanding of the social and cognitive boundaries among healthcare professionals and organizations [45, 48, 58], and (b) incorporate methods such as multi-level modelling, social network analysis and longitudinal and mixed methods studies, which involve some recognition and appreciation for the dynamic complexity of the healthcare system.

## About the authors

Dr. Peter Tsasis is an Associate Professor of Management jointly appointed to the School of Health Policy and Management, the School of Administrative Studies, and the York Institute for Health Research at York University. He is a Fellow with the American College of Healthcare Executives and serves on their regional Professional Development Committee.

Jenna M. Evans is a PhD Candidate in Health Services Research at the Institute of Health Policy, Management and Evaluation at the University of Toronto. She is also a Vanier Canada Graduate Scholar, a Certified Health Executive with the Canadian College of Health Leaders, and a Fellow with the Health System Performance Research Network.

Sue Owen is a Senior Manager in the National Healthcare Advisory practice at KPMG Canada. She is a Certified Health Executive and a Director on the National Board of Directors with the Canadian College of Health Leaders. She is also an Adjunct Professor at York University.

## Reviewers

**Carmel Martin,** PhD, Researcher, Northern Ontario School of Medicine, Sudbury, Ontario, Canada.

**Peter Nugus**, PhD, Fulbright Post-doctoral scholar, Department of Sociology, University of California Los Angeles, USA.

**Paul Williams**, PhD, School of Management, Cardiff Metropolitan University, Western Avenue, Cardiff, CF5 2YB, Wales.

**Mary E. Wiktorowicz,** Chair and Associate Professor, School of Health Policy and Management, HNES Building, 4th floor, York University, 4700 Keele Street, Toronto, Ontario M3J 1P3, Canada.

## References

[1] Singer S, Burgers J, Friedberg M, Rosenthal M, Leape L, Schneider E (2011). Defining and measuring integrated patient care: promoting the next frontier in health care delivery. Medical Care Research and Review.

[2] Grone O, Garcia-Barbero M (2001). Integrated care: a position paper of the WHO European office for integrated health care services. International Journal of Integrated Care [serial online].

[3] Ronson J (2006). Local health integration networks: will “made in Ontario” work?. Healthcare Quarterly.

[4] Ball T, Pointer D, Verlaan-Cole L (2004). Governance and management roles in transforming and integrating independent organizations within interdependent local health networks. Law and Governance.

[5] Ontario Health Quality Council (OHQC) (2010). 2010 report on Ontario’s health system.

[6] Thompson L, Martin M (2004). Integration of cancer services in Ontario: the story of getting it done. Healthcare Quarterly.

[7] Williams P, Lum J, Deber R, Montgomery R, Kuluski K, Peckham A (2009). Aging at home: integrating community-based care for older persons. Healthcare Papers.

[8] The Change Foundation (2008). Who is the puzzle maker? Patient/caregiver perspectives on navigating health services in Ontario.

[9] Health Quality Ontario (2011). 2011 report on Ontario’s health system.

[10] Bevan G, Janus K (2011). Why hasn’t integrated health care developed widely in the United States and not at all in England?. Journal of Health Politics, Policy and Law.

[11] Hudson B (2011). Ten years of jointly commissioning health and social care in England. International Journal of Integrated Care [serial online].

[12] Jiwani I, Fleury M (2011). Divergent models of integration: the Canadian way. International Journal of Integrated Care [serial online].

[13] Kodner D (2009). All together now: a conceptual exploration of integrated care. Health Care Quarterly.

[14] Shortell S, Gillies R, Anderson D, Mitchell J, Morgan K (1993). Creating organized delivery systems: the barriers and facilitators. Hospital and Health Services Administration.

[15] Leutz W (1999). Five laws for integrating medical and social services: lessons from the United States and the United Kingdom. The Milbank Quarterly.

[16] Burns L, Muller R (2008). Hospital-physician collaboration: landscape of economic integration and impact on clinical integration. The Milbank Quarterly.

[17] Ham C, Curry N (2010). Clinical and service integration: the route to improved outcomes.

[18] Hollander M, Prince M (2008). Organizing health care delivery systems for persons with ongoing care needs and their families: a best practices framework. Health Care Quarterly.

[19] Lega F (2007). Organisational design for health integrated delivery systems: theory and practice. Health Policy.

[20] Suter E, Oelke N, Adair C, Armitage G (2009). Ten key principles for successful health systems integration. Healthcare Quarterly.

[21] Shortell S, Gillies R, Anderson D (1994). The new world of managed care: creating organized delivery systems. Health Affairs.

[22] Friedman L, Goes J (2001). Why integrated health networks have failed. Frontiers of Health Services Management.

[23] Shortell S, Gillies R, Anderson D, Erickson K, Mitchell J (2000). Remaking health care in America the evolution of the organized delivery system.

[24] Browne G, Kingston D, Grdisa V, Markle-Reid M (2007). Conceptualization and measurement of integrated human service networks for evaluation. International Journal of Integrated Care [serial online].

[25] Browne G, Roberts J, Gafni A, Byrne C, Kertyzia J, Loney P (2004). Conceptualizing and validating the human services integration measure. International Journal of Integrated Care [serial online].

[26] Williams P, Sullivan H (2009). Faces of integration. International Journal of Integrated Care [serial online].

[27] Arndt M, Bigelow B (2008). Vertical integration in hospitals: a framework for analysis. Medical Care Review.

[28] Burns L, Pauly M (2002). Integrated delivery networks: a detour on the road to integrated health care?. Health Affairs.

[29] Ahgren B (2010). Mutualism and antagonism within organizations of integrated health care. Journal of Health Organization and Management.

[30] Edgren L (2008). The meaning of integrated care: a systems approach. International Journal of Integrated Care [serial online].

[31] Edgren L, Barnard K (2012). Complex adaptive systems for management of integrated care. Leadership in Health Services.

[32] Nugus P, Carroll K, Hewett D, Short A, Forero R, Braithwaite J (2010). Integrated care in the emergency department: a complex adaptive systems perspective. Social Science and Medicine.

[33] Waldrop M (1992). Complexity: The emerging science at the edge of order and chaos.

[34] Chaffee M, McNeill M (2007). A model of nursing as a complex adaptive system. Nursing Outlook.

[35] Holden L (2005). Complex adaptive systems: concept analysis. Journal of Advanced Nursing.

[36] Martin C, Sturmberg J (2009). Complex adaptive chronic care. Journal of Evaluation in Clinical Practice.

[37] McDaniel R, Driebe D, Blair JD, Fottler MD, Savage GT (2001). Complexity science and health care management. Advances in health care management.

[38] McDaniel R, Lanham H, Anderson R (2009). Implications of complex adaptive systems theory for the design of research on health care organizations. Health Care Management Review.

[39] Plsek P, Greenhalgh T (2001). The challenge of complexity in health care. British Medical Journal.

[40] Rowe A, Hogarth A (2005). Use of complex adaptive systems metaphor to achieve professional and organizational change. Nursing and Health Care Management and Policy.

[41] Sturmberg J, Martin C (2012). Understanding health system reform—a complex adaptive systems perspective. Journal of Evaluation in Clinical Practice.

[42] Tan J, Wen H, Awad N (2005). Health care and service delivery systems as complex adaptive systems. Communications of the ACM.

[43] Patton M (2002). Qualitative research and evaluation methods.

[44] Wallis S (2008). Emerging order in CAS theory: mapping some perspectives. Kybernetes.

[45] Ferlie E, Fitzgerald L, Wood M, Hawkins C (2005). The nonspread of innovations: the mediating role of professionals. Academy of Management Journal.

[46] Batalden P, Mohr J (1997). Building knowledge of health care as a system. Quality Management in Health Care.

[47] Druskat V, Pescosolido A (2002). The content of effective teamwork mental models in self-managing teams: ownership, learning and heedful interrelating. Human Relations.

[48] Evans J, Baker R (2012). Mental models of integrated care: aligning multiple stakeholder perspectives. Journal of Health Organization and Management.

[49] Begun J, Zimmerman B, Dooley K, Mick SM, Wyttenbach M (2003). Health care organizations as complex adaptive systems. Advances in Health Care Organization Theory.

[50] Lorenz E (1993). The essence of chaos.

[51] Donaldson C, Currie G, Mitton C (2001). Integrating Canada’s disintegrated health care systems: lessons from abroad.

[52] Leatt P, Pink G, Guerriere M (2000). Towards a Canadian model of integrated healthcare. Healthcare Papers.

[53] Mur-Veeman I, van Raak A, Paulus A (1999). Integrated care: the impact of governmental behaviour on collaborative networks. Health Policy.

[54] Kodner D, Spreeuwenberg C (2002). Integrated care: meaning, logic, applications, and implications—a discussion paper. International Journal of Integrated Care [serial online].

[55] Barnsley J, Lemieux-Charles L, McKinney M (1998). Integrating learning into integrated delivery systems. Health Care Management Review.

[56] Minkman M, Ahaus K, Huijsman R (2009). A four phase development model for integrated care services in the Netherlands. BMC Health Services Research [serial online].

[57] van der Linden B, Spreeuwenberg C, Schrijvers A (2001). Integration of care in the Netherlands: the development of transmural care since 1994. Health Policy.

[58] van Wijngaarden J, de Bont A, Huijsman R (2006). Learning to cross boundaries: the integration of a health network to deliver seamless care. Health Policy.

